# The Control of Movements via Motor Gamma Oscillations

**DOI:** 10.3389/fnhum.2021.787157

**Published:** 2022-01-17

**Authors:** José Luis Ulloa

**Affiliations:** Programa de Investigación Asociativa (PIA) en Ciencias Cognitivas, Centro de Investigación en Ciencias Cognitivas (CICC), Facultad de Psicología, Universidad de Talca, Talca, Chile

**Keywords:** neural oscillations, gamma, movement, motor cognition, conflict

## Abstract

The ability to perform movements is vital for our daily life. Our actions are embedded in a complex environment where we need to deal efficiently in the face of unforeseen events. Neural oscillations play an important role in basic sensorimotor processes related to the execution and preparation of movements. In this review, I will describe the state of the art regarding the role of motor gamma oscillations in the control of movements. Experimental evidence from electrophysiological studies has shown that motor gamma oscillations accomplish a range of functions in motor control beyond merely signaling the execution of movements. However, these additional aspects associated with motor gamma oscillation remain to be fully clarified. Future work on different spatial, temporal and spectral scales is required to further understand the implications of gamma oscillations in motor control.

## The Neural Organization of Movements

Human cognition is embedded in our interactions with others and with the environment (Varela et al., 2016; Rossi et al., 2019). From this point of view, to understand cognition is critical to understand how action and motor functions are realized within the nervous system. The impact of actions on our minds has been historically neglected, but now it has been acknowledged that even simple movements can modulate cognitive functions (Leisman et al., 2016). The motor system constitutes all those processes involving the movement of the muscles and the neural systems advocated to the control of the muscles. The motor system is tightly interconnected with other neural systems to organize movements. A quintessential example of this interaction occurs between perception and action systems (Prinz, 1997; Jeannerod, 2006). Perception and action are linked even at the lowest level of organization in the motor system, with feedforward and feedback loops being essential for motor control. The function of basic units in the motor system directly commanding the muscles depends on feedback from the muscles and from information in tendons and joints (Poppele and Bosco, 2003; Windhorst, 2007). This reciprocal interaction is replicated in higher levels of organization of the motor system. The brain circuits associated with movements converge on the primary motor cortex (M1 or Brodmann area 4) and signals are sent through the spinal cord to the muscles so motor commands are executed. However, previous to M1 activity the smooth unfolding of actions is programmed and organized in motor-related brain areas such as the premotor cortex (PMC, lateral part of the Brodmann area 6), the supplementary motor area (SMA, medial part of the Brodmann area 6) and the prefrontal cortex (PFC). The SMA forms a circuit loop with the PFC to organize voluntary actions devised in the PFC, while the PMC forms a circuit loop with parietal cortices and seems to be more reactive to externally evoked movements (Donoghue and Sanes, 1994). This network of brain regions extends to other subcortical areas such as the thalamus, the basal ganglia and the cerebellum, which contribute to the fine tuning of motor parameters in iterative loops that affect the ongoing activity of the muscles (Rosenbaum, 2010). Connections with high-level centers such as the PFC are supposed to feed with action “intentions” and “goals.” It is thus important to understand how these brain signals are transmitted to regions downstream in the motor system. In addition, human movements are not realized in a vacuum. Actions are created and modulated by a rich context, with motor programs being regulated and monitored at distinct levels of the motor system (Haggard, 2008). These distinct levels where action processing occurs have been investigated with modern brain imaging techniques. Among these methods, the electrophysiological techniques are useful for identifying and characterizing brain rhythms associated with action processing.

## The Neural Dynamics of Movements

Time-resolved electrophysiological techniques allow us to capture the macroscopic neural dynamics of the nervous system. This means we can precisely describe the sequence of neural activations that occur across multiple regions in the brain as humans or other animals engage in an activity or remain at rest. Rhythmic brain activity is associated with many perceptual and cognitive processes contributing to both low to high-level functions in humans (Varela et al., 2001; Buzsaki, 2006; Hari and Puce, 2017). In the motor system, the coding of movements has been shown to rely on the collective activity of a population of neurons (a population code; Georgopoulos et al., 1982, 1983). Likewise, rhythmic brain activity also seems to play an important – albeit not fully understood – role in the organization of movements (see van Wijk et al., 2012; Cheyne, 2013 for excellent reviews). Many studies investigating movement production have been focused on well-known neural oscillations in the alpha (10–12 Hz) and beta (13–30 Hz) band ranges. There is consistent evidence that execution of movements induces a decrease in alpha (Pfurtscheller, 1981; Salmelin and Hari, 1994; Cochin et al., 1999; Muthukumaraswamy et al., 2004; Neuper et al., 2006; Koelewijn et al., 2008) and beta (Cheyne et al., 2008; Gaetz et al., 2010; Wilson et al., 2010, 2014) oscillatory activity in sensorimotor cortices. Beta activity is characterized by building up well before the onset of movements, persisting and waning during the movement and being followed by a strong re-synchronization after movement end (beta rebound; Jurkiewicz et al., 2006; Engel and Fries, 2010; Zaepffel et al., 2013). More recently, gamma oscillatory activity (above 40 Hz) has attracted attention by its putative role in motor control. Gamma oscillations have been widely studied in vision and attention research, where they have shown a crucial role in integrating visual information (Gray and Singer, 1989; Tallon-Baudry et al., 1997). Based on these studies, it has been proposed that gamma oscillations may be a general cortical activity that integrates events among separated areas of the cortex for a variety of cognitive processes (Bressler, 1990; Fries, 2005). Based on recent works, researchers have started to uncover the functions of gamma oscillations in the motor system. In the following sections, I will outline distinct aspects of motor gamma oscillations, including local and long-range activity involved in simple and complex movements. The reader can also consult a summary table at the end of the manuscript that I have prepared with methodological details and main findings.

## Motor Gamma Oscillations

### Overview

In a seminal study using electrocorticography (ECoG) Crone et al. (1998) identified two kinds of motor gamma responses. A motor gamma centered in the 35–50 Hz range that started with the motor response and remains active during the duration of the movement and a motor gamma activity centered in the 75–100 Hz range that started slightly before the motor response and was transient. This latter neural activity was somatotopically organized and was more circumscribed at contralateral sides than alpha or beta oscillations. Follow-up studies have confirmed these findings for a wide range of gamma frequency bands using again ECoG (Pfurtscheller et al., 2003; Leuthardt et al., 2004; Ball et al., 2008; Darvas et al., 2010), but also using magnetoencephalography (MEG; Dalal et al., 2007; Cheyne et al., 2008; Tecchio et al., 2008; Muthukumaraswamy, 2010; Wilson et al., 2010; Trevarrow et al., 2019; Spooner et al., 2021; Wiesman et al., 2021), scalp electroencephalography (EEG; Pfurtscheller et al., 1993; Ball et al., 2008; Darvas et al., 2010; Herz et al., 2012; Oliveira et al., 2019; Djalovski et al., 2021) and stereo EEG (Brovelli et al., 2005; Szurhaj et al., 2005). The detected gamma activities in EEG tend to be in the low frequency band range near 40 to 60 Hz because of the filtering properties of the scalp, cortical orientations of neuronal populations and other factors that limit the detection of fast rhythms in EEG (Nunez and Srinivasan, 2006). The range of frequencies in which motor gamma has been found is quite variable, analogous to what happens in gamma oscillations in other domains (Uhlhaas et al., 2011). In the motor domain, gamma oscillations have been reported for ranges between 75–100 Hz (Crone et al., 1998), 65–90 Hz (Dalal et al., 2007), 59–84 Hz (Ball et al., 2008), 81–101 Hz (Darvas et al., 2010), 60–90 Hz (Muthukumaraswamy, 2010; Grent-’t-Jong et al., 2013), 64–84 Hz (Wiesman et al., 2020), and 72–84 Hz (Spooner et al., 2021), to name a few. In intracranial EEG studies, the range of motor gamma is even broader, covering a 60–200 Hz range (Brovelli et al., 2005). These distinct frequency ranges may also reflect distinct forms of gamma oscillations in the high frequency range (Uhlhaas et al., 2011). To date, there is no clear understanding of the functional significance of distinct gamma types. To develop a full picture of gamma oscillations, efforts should be done to systematically and precisely describe the frequency range in which motor gamma oscillations occur.

Motor gamma oscillations have been observed for simple movements, including finger movements, tongue protrusions, eye-winking, fist-clenching and foot movements (Pfurtscheller and Neuper, 1992; Crone et al., 1998; Pfurtscheller et al., 2003; Miller et al., 2007; Cheyne et al., 2008), but they have been also observed for more elaborated representations of actions such as motor imagery (Miller et al., 2010; Grosse-Wentrup et al., 2011), mirroring of movements (Butorina et al., 2014), walking and cycling movements (Gwin et al., 2011; Seeber et al., 2016) and interpersonal interaction (Djalovski et al., 2021). Motor gamma oscillations seem also to change during human development. It has been reported that gamma oscillations associated with motor processing are very variable in frequency for very young children, with frequency ranges varying between 35–45 and 70–80 Hz (Cheyne et al., 2014). This variability seems to settle in 70–80 Hz in children, adolescents and adults (Gaetz et al., 2010). In addition, the power of motor gamma oscillations has been reported to change from childhood to adolescence, with decreases of gamma oscillations at the M1 (Trevarrow et al., 2019) and the SMA (Wilson et al., 2010). One interpretation of these findings is that, as the nervous system matures, motor control becomes more localized and efficient (Wilson et al., 2010). The reported decrease of gamma oscillations in adolescence contrast with another study showing an increase of motor gamma power from childhood to adolescence and weaker motor gamma in adults relative to adolescents (Gaetz et al., 2010). There is also evidence that lateralization of motor gamma activity is modulated during adolescence (Huo et al., 2011), meaning that the strong lateralization of motor gamma oscillations is the product of a developmental trajectory. Overall, these studies highlight the pervasive nature of gamma oscillations in distinct forms of motor processing and its changes during development.

### Local Gamma Activity

It has been often remarked that brain oscillations at the lower end of the spectrum tend to engage large areas, while those oscillations at higher frequencies are localized in restricted cortical areas (Lopes da Silva, 2013). Local motor gamma activity has been generally found in M1 (Crone et al., 1998; Cheyne et al., 2008; Darvas et al., 2010; Muthukumaraswamy, 2010), but also from accessory motor regions in the SMA (Szurhaj et al., 2005; Ball et al., 2008; Wilson et al., 2010; Tamás et al., 2018), the PMC (Brovelli et al., 2005; Gaetz et al., 2013; Dürschmid et al., 2014; Wiesman et al., 2020) and regions closer to the frontal cortex (Gaetz et al., 2013; Grent-’t-Jong et al., 2013). In addition, it has been widely recognized that M1 receives inputs from subcortical areas such as the thalamus, the cerebellum, and the basal ganglia. The basal ganglia is a set of interconnected nuclei in the forebrain that exerts an inhibitory influence on several motor systems (Nambu et al., 2002). Motor gamma activity has been also found in the basal ganglia, in particular in the globus pallidus (GB) and the subthalamic nucleus (STN; Alegre et al., 2005; Brücke et al., 2008). This broad motor gamma network is potentially critical for the organization of movements. However, the relationship between gamma oscillations arising from cortical and subcortical regions is still poorly understood. Hints of a possible relationship have been suggested. A study designed to compare motor gamma activity evoked for upper and lower limbs found that the peak frequency of gamma was slightly higher, and consistent within individuals, for both right and left fingers and elbows relative to foot movements (Cheyne et al., 2008; Cheyne and Ferrari, 2013). These individual gamma “fingerprints” led the authors to speculate that gamma activity detected at cortical sites may have a common origin and that source could be at the level of cortico-subcortical networks. Overall, findings from studies focusing on local neural activity show that gamma oscillations are distributed across several cortical and subcortical structures.

The nature of gamma oscillations is an ongoing mystery. Unlike neural oscillations in the low frequency range, like alpha and beta, motor gamma oscillations have been described as prokinetic, i.e., to promote movements. The increase of gamma oscillatory activity has been found to occur very close to triggering of movements (Crone et al., 1998; Pfurtscheller et al., 2003; Cheyne et al., 2008; Darvas et al., 2010; Muthukumaraswamy, 2010; but see also Muthukumaraswamy, 2010 for modulations at the movement end). This temporal feature suggests a role of gamma oscillations in the driving of movements, but the evidence is not clear. Some authors have suggested that motor gamma activity is associated with the processing of sensory reafferences (from the muscles) in sensory and motor cortical centers (Szurhaj et al., 2005). This view holds that motor gamma oscillations bind sensorimotor information and facilitate movement. In this sense, gamma oscillations should be strongly related to the processing of sensory information. However, in a MEG study, Muthukumaraswamy (2010) shows that motor gamma occurred equally well for both self-paced and evoked movements but not for passive movements. In this same study, the author also showed that motor gamma activity peaked only at the beginning of a sequence of repetitive movements and remained silent during the execution period. The short-lived behavior of motor gamma oscillations has been observed in other studies (Cheyne et al., 2008; Gaetz et al., 2013; Wiesman et al., 2020). Thus, motor gamma oscillations are tightly locked to the initiation of voluntary movements and they don’t seem to be a direct consequence of sensory feedback produced by movements. Using a mirror illusion effect, a study supports this idea. The illusion of a moving hand can be evoked through the reflection (in a mirror) of the movement of the opposite hand. Using this paradigm (Butorina et al., 2014) demonstrated an increase of motor gamma in a mirror manipulation. The increase of motor gamma oscillations in this illusion suggest that gamma activity is independent of proprioceptive feedback. It is worth noting, however, that gamma activity evoked during this illusion was less strong than the activity evoked for the actual movements. More studies will be required to clarify the contribution of sensory feedback to motor gamma activity. A role of motor gamma oscillations in the control of movements should also involve the coding of basic motor parameters. There is evidence that gamma oscillations are associated with the coding of motor parameters like force and direction. Muthukumaraswamy (2010) showed that greater motor gamma power at M1 was associated with greater force of movements. In addition, ECoG studies have shown that motor gamma power at the M1 could be used for decoding the direction of movements (Leuthardt et al., 2004; Ball et al., 2009; Yanagisawa et al., 2012). Interestingly, there are also studies showing that subcortical gamma activity is involved in the coding of basic of motor parameters associated with movements. Gamma activity at the STN is greater when greater force is required (Tan et al., 2013; Alhourani et al., 2020) and it is associated with larger (Brucke et al., 2012; Lofredi et al., 2018) and faster movements (Brucke et al., 2012; Joundi et al., 2012). Altogether, the current evidence highlight some of the characteristics of motor gamma oscillations: its close connection (at least temporally) with movement initiation and its contribution to the coding of some basic motor properties. However, recent works have acknowledged a more sophisticated role of motor gamma oscillations in the processing of actions.

In real life, movements need to be performed under challenging conditions or might entail rapid adjustments. Cognitive control describes the ability to generate, maintain and adjust strategies directed to specific goals, which altogether allows the emergence of flexible behavior (Botvinick et al., 2001). A key question in cognitive neuroscience has been how cognitive control is carried out in the brain. Distinct lines of research have emphasized the idea that cognitive control, and more generally, executive functions, are commanded by the PFC (Toba et al., 2020). In this line, electrophysiological evidence shows that these prefrontal operations involve theta (Cohen et al., 2008; Nigbur et al., 2011; Gulbinaite et al., 2014) and gamma (Jensen et al., 2007; Roux et al., 2012) oscillatory responses. However, emergent evidence suggests that gamma oscillations at M1 (Isabella et al., 2015; Heinrichs-Graham et al., 2018; Spooner et al., 2021), motor regions like the PMC (Gaetz et al., 2013; Wiesman et al., 2020) and medial frontal regions (Grent-’t-Jong et al., 2013) contribute to the neural processing of movements in the context of interference or conflict. These studies are based on cognitive psychology paradigms where participants are required to perform movements under conditions of interference and require the inhibition of responses. These studies have shown that motor gamma responses increase during interference in an Eriksen flanker (Grent-’t-Jong et al., 2013; Heinrichs-Graham et al., 2018; Spooner et al., 2021). In this task, participants respond to a central letter or object flanked by distractor stimuli that evoke an alternative response (Eriksen and Eriksen, 1974). In an incompatible or interference condition, a conflict is generated because a target attribute is presented alongside a distractor. To succeed in this task, participants have to focus on suppressing an automatic tendency to respond to the irrelevant dimension. Interestingly, in distinct versions of this task, there have been selective changes either in power (Grent-’t-Jong et al., 2013; Spooner et al., 2021) or the frequency (Heinrichs-Graham et al., 2018) of gamma oscillations. These differences could be due to differences in the experimental setting, such as the use of distinct button responses systems and the involvement of a different number of fingers. It is likely that our understanding of the impact of gamma oscillations in this type of motor responses will improve with more studies that incorporate distinct spectral metrics (power, frequency and phase). Motor gamma responses have been also studied in a modified Go/No-Go task where a switch condition is included (Isabella et al., 2015). In the Go/No-Go task, participants must perform speeded responses in Go trials and must refrain from responding on No-Go trials (Logan et al., 2014). In the No-Go condition, a response inhibition is used to cancel an intended movement. Conversely, in a switch condition, participants are asked to perform a distinct movement. In the study of Isabella et al. inhibition to stop was reflected in increased theta power and inhibition to switch was reflected in increased gamma power. These findings show a complementary roles of theta and gamma oscillations in the inhibition of responses (Isabella et al., 2015). Gamma activity is also modulated by contextual information. In another MEG study, participants were asked to perform a repetitive bimanual response in response to visual stimuli. For a given hand, the context was uncertain because in 20% of the trials a signal indicated that no movement has to be performed. For the other hand, the context was certain because there was never a stop signal and movements has to be executed every time (Wiesman et al., 2021). When contralateral responses of each hand were compared to each other the authors found increased motor gamma power for movements performed in the uncertain relative to the certain context. The engagement of motor gamma oscillations in this situation speak of mechanisms oriented to process the dynamic and uncertain conditions of our environment. These findings converge with other studies that show the responsiveness of motor gamma activity to environmental cues, such as in attentional capture (Spooner et al., 2020). This responsiveness to dynamical environmental demands highlights the adaptive functions that motor gamma oscillations have and its potential implications for our interactions with our surrounding environment. Lastly, it is worth mentioning that subcortical gamma activity has been also observed in experimental situations where participants are required to inhibit responses. There is robust evidence of the involvement of the basal ganglia in motor control. A basal ganglia circuit involves close interactions with cortical and other subcortical regions and is important for fine adjustment of movements and the inhibition of responses. This circuit has two main pathways. A direct pathway has been associated with facilitation of movement preparation, while an indirect pathway has been associated with the suppression of movement preparation (Chakravarthy, 2013). In this basal ganglia circuit the STN operate as a break within the indirect pathway. In addition, there is a hyperdirect pathway (a faster route than the aforementioned pathways) where frontal (IFG, PFC) and motor (preSMA) regions are connected directly with the STN (Swann et al., 2012; Chen et al., 2020). This pathway seems to be important for the inhibition of automatic responses. In these studies, the inhibition of responses can be assessed with a Stop signal task. In this task, a participants had to respond to a visual stimulus (go cue) and in a set of trials this go cue is followed by a stop signal. The task is to stop the ongoing response to the cue (Bari and Robbins, 2013). Recent work has demonstrated that gamma activity at the STN is modulated during inhibitory responses (Ray et al., 2012; Alegre et al., 2013; Fischer et al., 2017). Altogether the evidence reviewed above suggest that local gamma activity at cortical and subcortical areas is important for distinct types of movements and for the inhibition of responses. Beyond local activity, gamma oscillations have been also hypothesized to carry information in structures far away apart.

### Brain-Muscle Coupling

Researchers have been dealing for long time to understand how motor commands are transmitted from the cortex to the muscles. Axons from the neurons in high-level processing areas of the motor system descend through the spinal cord and reach the neurons that connect directly with the muscles (Rosenbaum, 2010). In the spinal cord, alpha neurons connecting with the muscles form what has been called motor units. Each motor unit is composed of a motor neuron and all the muscles this motor neuron innervates (Rosenbaum, 2010). The electrical activity of the muscles indirectly reflects the activity of spinal alpha neurons (and thus of motor units) and is measured by electromyography (EMG). The study of muscular activity originated from early investigations by Hans Piper, who detected a muscular 40 Hz activity using a stethoscope. This rhythm is called the Piper rhythm in his honor. In a seminal study in humans, Conway et al. (1995) demonstrated that brain signals show brain-muscle coupling (as coherence) in the beta range when participants are asked to perform isometric contractions with weak force. Isometric contractions are static compressions of the muscle and occur without movement. Later studies showed that a contraction entailing force may lead to a decrease of beta coherence (Kilner et al., 1999) and a predominance of coherence in the gamma range (∼40 Hz; Brown et al., 1998; Mima et al., 1999; Li et al., 2020). Gamma brain-muscle coupling is produced also for slow movements (Salenius et al., 1996; Marsden et al., 2000), repeated maximal contractions (Brown et al., 1998) and isotonic movements (for lower limb movements; Gwin and Ferris, 2012). Unlike isometric contractions, isotonic movements are associated with changes in the muscle’s length. Some studies pinpoint to some distinctions in the brain muscle-coupling in the gamma or beta range. For instance, Omlor et al. (2007) showed that gamma coupling occurs in complex or dynamic movements, while beta is predominant for movements with a stable motor output. It has been also hypothesized that gamma coupling could be important for the correct prediction of errors in fast force transitions (Mehrkanoon et al., 2014). Finally, local gamma activity seems to be independent of brain-muscle coupling. Muthukumaraswamy (2011) showed that for simple and repetitive movements, cortical gamma increase was paralleled by an increase of brain-muscle coupling, but for static force production there is a burst of gamma activity without an increase in brain-muscle coupling. These findings suggest different functional processes reflected in brain-muscle coupling and cortical activity.

There is a more general issue that involves the synchronic nature of brain-muscle coupling. Some studies have found instantaneous (or zero delay) synchronization in brain-muscle coupling (Conway et al., 1995; Halliday et al., 1998). In the motor system, a mechanism that could induce zero delay synchronization could involve afferent feedback from the muscles. There is evidence supporting this notion. For instance, ischemia-induced deafferentation (producing lack afferent feedback) dampen brain-muscle coupling (Pohja and Salenius, 2003). Conversely, it has been shown that affecting spindle activity in the muscles does not change the Piper rhythm (Hagbarth et al., 1983) or the brain-muscle coupling (Mima et al., 2000). In addition, there are studies reporting a delay between brain and the muscle signals in brain-muscle coupling (Salenius et al., 1997; Brown et al., 1998; Mima et al., 2000). This lag occurs in a direction where the motor cortex is activated first and drives the activity of the muscle. The sources of the leading activity in the brain have been shown to follow a somatotopic organization and to be at the M1 (Gross et al., 2005), although the SMA may also contribute (Salenius et al., 1997; Hari and Salenius, 1999). It has been also reported a 15 ms longer delay in brain-muscle coupling for lower relative to upper limb movements (Mima et al., 2000). These differences have been in part explained as differences in the conduction distance between the cortex and the muscle. The practical importance of brain-muscle coupling to motor behavior is still discussed. Some studies have shown an increased brain-muscle gamma coupling with increased readiness to respond in simple reaction time experiments (Schoffelen et al., 2005, 2011). There remain several aspects of motor-muscle coupling about which relatively little is known.

### Brain–Brain Coupling

It is currently acknowledged that neural functions derive from some specialization of brain areas. However, the modern view maintains that cognitive functions emerge from the cooperative participation of groups of brain regions or networks (Sporns, 2011). While local neural activity covers an area of ∼1 cm through monosynaptic connections, large-scale activity is said to occur between neural assemblies that are over 1 cm apart and involve polysynaptic pathways (Varela et al., 2001). Long-range interactions involve a complex configuration of connections between distinct nodes that are difficult to understand. This is further complicated by the fact that connectivity measures show limitations because of the probability of spurious synchronization. The exact functional significance of brain–brain coupling has not been demarcated, but it is assumed that the co-activation of brain regions during a task reflects the relevance of a given neuronal ensemble for the cognitive process under study. In the motor domain, brain regions related to action processing are thought to act in concert so precise and smooth movement are brought about. Studies of connectivity have been propelled by former functional magnetic resonance and positron emission tomography that defined the key nodes associated with the motor brain network. For instance, simple finger movements involved a lateralized motor network comprising regions like the M1, PMC and the SMA (Catalan, 1998; Diciotti et al., 2007). Electrophysiological studies have investigated the oscillatory coupling between these regions (e.g., Gerloff, 1998; Gross et al., 2005). For instance, Gross et al. (2005) investigated with MEG the connectivity across several frequency bands for a set of unimanual and bimanual movements. This study showed that left M1-right M1 coupling was greater for bimanual relative to unimanual movements. In addition, it was shown that changes in gamma M1-SMA coupling and gamma brain-muscle coupling occurred for movements with distinct complexity. This basic core motor network has been naturally extended to other regions, like the PFC and posterior parietal cortex. For instance, it has been shown that distinct types of finger movements involve distinct gamma connectivity profiles between primary sensorimotor cortices with the SMA and the PFC (Tamás et al., 2018). Differences in patterns of connectivity has been also disclosed with dynamic causal modeling. In an EEG study, it has been shown that isometric contractions of the forearm involve gamma coupling between the M1 and SMA, while repetitive finger movements involve an additional coupling between the PMC and both the M1 and SMA (Herz et al., 2012). Again, subcortical regions have been seen to contribute to motor control and to code basic motor parameters. For instance, it has been shown increased cortico-subcortical coupling between the M1 and STN for increased force (Alhourani et al., 2020) and velocity (Fischer et al., 2020) in manual responses. Gamma coupling has been also seen in responses associated with inhibition. For instance, an ECoG showed increased gamma coupling between the preSMA and right inferior frontal gyrus (IFG) for responses that required a stopping response (Swann et al., 2012).

Brain–brain coupling can also occur across different frequency bands. Typically, activities in the lower frequency band modulate the amplitude, frequency, or phase of the higher frequency signal. This cross-frequency coupling (CFC) has been linked to several cognitive processes and has been shown to be altered in pathological states (Canolty and Knight, 2010). A form of CFC, phase-amplitude coupling (PAC) has been typically found between theta and gamma, where the phase of theta activity modulates the amplitude of gamma activity (Lisman and Jensen, 2013). Theta-gamma coupling has been also shown in the motor domain. A study involving a variety of distinct tasks (a serial response, auditory motor coordination and Go/No-Go task) revealed that an increase theta-gamma PAC coupling is associated with an increment in motor performance (Dürschmid et al., 2014). These effects reflect a role of motor gamma oscillations in association with theta rhythms in motor learning. PAC coupling has been also shown to be involved in inhibition of responses. In an MEG study, participants were asked to perform a social approach-avoidance task where they have to avoid or approach emotional displays using a joystick (Bramson et al., 2018). The authors observed that theta power at the PFC was modulated by the congruence conditions, with increased theta activity in the incongruent relative to the congruent condition. Cognitive control exerted by the PFC was further qualified by PAC between PFC theta and M1 motor gamma. This is, the increase of gamma activity during emotional action control was modulated by the PFC. Even more, in a transcranial alternating current stimulation (tACS) study, emotional control was elicited by increasing this PFC-M1 theta-gamma coupling (Bramson et al., 2020). Other studies have characterized theta-gamma coupling and found that is modulated by sensory modulations. Somatosensory entrainment reduced theta-gamma PAC in simple fingers movements in a visual response paradigm (Spooner et al., 2021). The evidence presented support the idea that gamma activity occurs locally and in large-scale interactions. From a functional point of view, motor gamma encodes certain basic motor parameters and seems to play a role in inhibitory responses. More evidence still needs to be gathered to fully understand the role of gamma oscillations in movements. Another important aspect of gamma is its biological substrates. This is important since it has been observed that disturbances in the generation of gamma oscillations have been associated with some neurological and psychiatric conditions.

### Neurobiological Mechanisms and Physiopathology

Neural oscillations correspond to rhythmic fluctuations in the excitability of populations of neurons occurring at distinct spatial and temporal scales (Varela et al., 2001; Buzsaki, 2006). The mechanisms that give rise to oscillations involve the interactions between inhibitory interneurons, based on aminobutyric acid (GABA)ergic neurotransmission, and excitatory pyramidal cells, based on glutamatergic neurotransmission. An alternating cycle between excitatory and inhibitory states emerges when excitatory pyramidal cells get activated and stimulate interneurons which then inhibit the excitatory cells. The following decrease in inhibition allows the excitation period to start again (Buzsáki and Wang, 2012). Both excitatory and inhibitory mechanisms are important for the generation of neural oscillations, particularly in the gamma band. Excitatory mechanisms led by α-amino-3-hydroxy-5-methyl-4-isoxazolepropionic acid (AMPA) and N-methyl-D-aspartate (NMDA) neurotransmission is critical for the generation of gamma oscillations (Fuchs et al., 2001; Carlén et al., 2012). Similarly, the inhibitory features of parvalbumin-expressing GABAergic interneurons are a key element in the generation of gamma oscillations (Sohal et al., 2009). The role of GABA neurotransmission has been investigated with MEG and pharmacological interventions. Some studies have shown that modulations of GABA neurotransmission are more directly associated with changes in beta rather than gamma oscillations in the motor cortex (Gaetz et al., 2011; Hall et al., 2011; Muthukumaraswamy et al., 2013). However, enhancing gamma activity with transcranial magnetic stimulation (TMS) has been associated with changes in GABA levels (Nowak et al., 2017). Different outcomes from these studies could be related to distinct methods to measure GABA and to differences in the used paradigms. For instance, while Nowak et al. (2017) applied a Go/No-Go task, the aforementioned studies use simple reaction time paradigms. Besides neurotransmission, gamma oscillations also emerge as a result of network properties, such as mutual inhibition, mutual excitation and recurrent inhibition (Uhlhaas et al., 2011). These properties define models where gamma oscillations arise from locally generated excitations or inhibitions within an ensemble, or could result from increased input (Sedley and Cunningham, 2013). The current evidence highlights the importance of neurotransmission systems and network properties for the emergence of gamma oscillations. In line with a role of excitatory and inhibitory states in the normal brain functioning, unbalanced neurotransmission and alterations of oscillatory activity have been associated with neuropsychiatric and neurological disorders.

Schizophrenia is a complex neuropsychiatric disorder characterized by delusions, hallucinations, apathy and a loss of social motivation. It has been shown that patients with schizophrenia have aberrant gamma oscillations in perceptual (Uhlhaas et al., 2006) and executive (Cho et al., 2006) functions. More recently, it has been also shown that these patients exhibit motor disturbances and these alterations may involve changes in motor oscillations. Compared with healthy individuals, early onset schizophrenic patients show a reduction of gamma oscillatory responses in M1 and the cerebellum in a finger movement paradigm (Wilson et al., 2011). These findings suggest that alterations of gamma activity span distinct cognitive domains and may reflect a general dysfunction of gamma oscillatory activity. This notion converges with the idea of an altered neurotransmission balance in schizophrenia (Gaspar et al., 2009). Autism spectrum disorder (ASD) is a neurodevelopmental disorder characterized by deficits in social communication, restricted interests, and repetitive behaviors (American Psychiatric Association, 2013). In a series of studies with MEG and a game-like motor task An et al. (2018, 2021) investigated the neural responses associated with finger presses in normal and ASD children. These studies showed a decrease in gamma peak activity (An et al., 2018) and decreased beta-gamma ipsilateral PAC activity (An et al., 2021) in M1 of ASD children in comparison with healthy participants. Once again, these modulations of motor gamma oscillations may reflect an unbalance in the excitatory and inhibitory neurotransmission systems. Indeed, these neurotransmissions anomalies have been hypothesized to be a core aspect of ASD (Rubenstein and Merzenich, 2003). Lastly, Parkinson’s disease is a neurodegenerative disorder leading to tremor, bradykinesia, stiffness, and difficulty with walking, balance, and coordination. PD is characterized by a loss of nigrostriatal dopaminergic neurons and thus dopamine replacement has been used as a pharmacological treatment (Lang and Lozano, 1998a). A more recent therapy is based on deep brain stimulation (DBS) of the STN and has been shown to decrease motor disturbances in PD (Lang and Lozano, 1998b). Interestingly, improvement of motor functions seems to be related to a decrease of beta oscillations (which are over activated in the disorder) and an increase of motor gamma oscillations (Brown et al., 2001). These findings highlight the complementary roles of beta and gamma oscillations during health and a how an alteration of the oscillatory activities might underlie pathological conditions.

Gamma oscillations are modulated by alterations in the normal functioning of the nervous system that compromise the production of movements. A stroke occurs when the blood supply to a brain region is impeded (possibly by an obstruction or a rupture of the vasculature). Stroke can lead to sensory deficits problems to produce or understand speech and is associated with impairments in motor functions. It has been shown that patients with stroke have decreased brain-muscle gamma coupling than healthy controls during the realization of movements (Fang et al., 2009; Rossiter et al., 2013). Interestingly, peripheral nerve stimulation therapy in patients with stroke shows decreased M1 gamma activity and these effects are related to an improvement in motor symptoms (Wilson et al., 2011). These effects are said to result from a more efficient local processing in the motor cortex after therapy. Cerebral palsy (CP) is a common motor disability in children that affects the ability to move and maintain the balance of the body. Studies show an aberrant gamma response in children with CP compared with healthy individuals, either with an increase (Guo et al., 2012; Short et al., 2020) or decrease (Kurz et al., 2014; Hoffman et al., 2019) of gamma activity. The reported gamma abnormalities in CP span distinct gamma ranges, with intervals between 38 and 56 Hz to interval between 70 and 200 Hz. However, studies differ in disorder specificities, brain recording methods, as well as in using distinct experimental manipulations, including walking (Short et al., 2020), simple bodily responses (Kurz et al., 2014), response inhibition (Hoffman et al., 2019) and electric stimulation (Guo et al., 2012). Overall, these cases support the view that abnormalities in motor gamma oscillations are implicated in neuropsychiatric and neurological disorders.

## Future Directions

I have tried to summarize our current understanding of the role of motor gamma oscillations in the control of movements. There are many aspects of motor gamma oscillations that need to be clarified in future studies. Still, the current evidence suggests that motor gamma oscillations accomplish a role beyond mere movement initiation. Local and long-range motor gamma activity serves as a core mechanism to organize distinct forms of motor processing. The impact of gamma motor oscillations probably relies on the extensive network of brain regions that include cortical, subcortical and spinal cord activity (indirectly reflected in the EMG measurement). The current challenge is to put all this in one or several models of motor control. There are many open questions and future directions that need to be taken. Research in this domain will be enriched with longitudinal studies, which will allow us to have a better grasp of the development trajectory of motor oscillations and whether and how these changes are contingent with changes in motor milestones in human beings (see Stephen et al., 2021 for an example). This endeavor will be only approachable within an interdisciplinary and collaborative framework. This is also important because “movement” data can take a variety of forms. A better interpretation of these data requires the collaboration between scientists from distinct disciplines. In this same line, it is necessary to replicate findings for a set of well-defined experimental paradigms with well-characterized movements. There are many types of movements reported in the literature. Both simple and complex types of movements should be systematically studied and can become potential markers for the use in clinical as well as more fundamental research on motor control. Gamma oscillations involve a wide range of brain regions beyond being confined to cortical regions. The versatility of gamma oscillations in the organization of movements is probably because of this wide network activity. A multi-scale approach to understanding brain connections that occur at distinct levels of processing will be essential. It is important to understand the specific contribution of each node in the sensorimotor gamma network, including primary sensorimotor cortices, as well the PMC, the SMA, and other regions, such as the parietal cortex and the PFC. The contribution of subcortical regions such as the thalamus, cerebellum, and basal ganglia needs to be integrated in the often cortico-centered models of motor control. There is robust evidence that the basal ganglia exerts an important influence on the organization of simple and complex responses. It is necessary to understand how the activity of the PFC influences downstream motor pathways. The ability to prepare actions and predict the consequences of our actions rely in a complex organization likely commanded by the PFC. In this line, new ideas about cognition and brain function suggest that the brain could operate as a Bayesian inference machine (Friston, 2010). In this framework, predictions derived from the internal models are confronted with new sensory information from the environment (a sensory consequence of a movement) and the comparisons between these signals update an internal model which subsequently can alter motor activity. Recent evidence suggest that neural oscillations associated with motor control in the beta (Tan et al., 2016) and gamma (Wiesman et al., 2021) range are sensitive to certainty of events in the external environment.

There are methodological aspects that deserve more attention. We need to better understand the role of different frequency gamma ranges and why there is such a frequency diversity in which gamma responses are found. One of the difficulties in the study of motor, and more generally, gamma oscillations, is its frequency variability. Using a diversity of neural metrics, future studies should aim to categorize the diversity of frequency ranges of gamma responses found in distinct brain areas. It is possible that the integrative properties endowed by gamma oscillations operate at distinct spatio-temporo-spectral dimensions. In addition, future studies will need to expand analyses methods to consider the complexity and non-linear nature of local and long-range brain activity. The inherent complexity of brain networks has not been fully explored, and can make up another dimension in which motor parameters are coded. We need also consider the social context in which movements are carried out. In models of motor control, it is necessary to incorporate the fact that the actions are carried out in a social context associated with social inter-actions. These interactions shape our experience and our perceptual processes. Future lines of research should be concerned with evaluating actions in both laboratory contexts and real contexts outside the laboratory. Flexible and adaptive behavior is grounded in our ability to perform actions, and this adaptive behavior varies according to the contingencies we encounter in our social daily life. Lastly, gamma oscillations are not the only oscillations involved in motor processing. In fact, it is intriguing that so many distinct oscillatory responses contribute to the processing of motor information in the nervous system. This attests to the importance of actions for human beings. The science of movements has become one of the most exciting challenges in neuroscience emerging these recent years. The evolution of this field has benefited from recent advances in experimental psychology, cognitive neuroscience and computational modeling, which altogether have dramatically sped up our understanding of movements. A collective effort will be instrumental to fully understand the role of motor gamma oscillations in human movements.

## Author Contributions

The author made substantial contributions to the discussion of content, wrote the article and edited the manuscript before submission.

## Conflict of Interest

The author declares that the research was conducted in the absence of any commercial or financial relationships that could be construed as a potential conflict of interest.

## Publisher’s Note

All claims expressed in this article are solely those of the authors and do not necessarily represent those of their affiliated organizations, or those of the publisher, the editors and the reviewers. Any product that may be evaluated in this article, or claim that may be made by its manufacturer, is not guaranteed or endorsed by the publisher.

## Supplementary Material

Supplementary Table 1 Summary of the studies associated with motor gamma oscillations included in the revision. The table depicts the reference; the method; the task or type of movement executed; the number of participants (N); the body part concerned with the movement; the frequency band range; the area or electrodes where gamma activity was found and the main findings. In “N” two or more numbers indicate distinct set of participants. In clinical studies “p” stand for patient and “h” stand for healthy participant. In “Frequency” the numbers indicate the frequency range (minimum maximum). Two or more numbers indicate distinct frequency ranges reported. STN, subthalamic nucleus; GP, globus pallidus; PAC, phase-amplitude coupling; IFG, inferior frontal gyrus; PFC, prefrontal cortex; M1, primary motor cortex; PMC, premotor cortex; SMA, supplementary motor cortex; DBS, deep brain stimulation; EEG, electroencephalography; MEG, magnetoencephalography; ECoG, electrocorticography; CMC, cortico-muscular coherence.
