# Supplementary material for: The Control of Movements via Motor Gamma Oscillations
Source: Front Hum Neurosci. 2022 Jan 17;15:787157. doi: 10.3389/fnhum.2021.787157 (PMC8802912; doi:10.3389/fnhum.2021.787157)
Supplement: Supplementary Table 1 — Summary of the studies associated with motor gamma oscillations included in the revision. The table depicts the reference; the method; the task or type of movement executed; the number of participants (N); the body part concerned with the movement; the frequency band range; the area or electrodes where gamma activity was found and the main findings. In “N” two or more numbers indicate distinct set of participants. In clinical studies “p” stand for patient and “h” stand for healthy participant. In “Frequency” the numbers indicate the frequency range (minimum maximum). Two or more numbers indicate distinct frequency ranges reported. STN, subthalamic nucleus; GP, globus pallidus; PAC, phase-amplitude coupling; IFG, inferior frontal gyrus; PFC, prefrontal cortex; M1, primary motor cortex; PMC, premotor cortex; SMA, supplementary motor cortex; DBS, deep brain stimulation; EEG, electroencephalography; MEG, magnetoencephalography; ECoG, electrocorticography; CMC, cortico-muscular coherence. [file Table_1.docx]

| **Reference** | **Method** | **Task/Movement type** | **N** | **Body** | **Frequency** | **Area** | **Main finding** |
| --- | --- | --- | --- | --- | --- | --- | --- |
| Alegre et al. 2005 | DBS, EEG | Wrist extensions | 6 | Hand | 70 80 | STN | Increased gamma power at the STN for stopping behavior |
| Alhourani et al. 2020 | DBS, EEG | Go/No-Go task | 10 | Hand | 60 150 | M1, STN | Gamma power correlates with movement force |
| An et al. 2018 | MEG | Finger presses | 14p, 15h | Finger | 60 100 | M1 | Decreased gamma peak activity is associated with autism symptoms |
| An et al. 2021 | MEG | Finger presses | 18p, 19h | Finger | 60 100 | M1 | Decreased ipsilateral beta-gamma PAC in autism |
| Ball et al. 2008 | EEG, ECoG | Arm reaching movements | 8 | Arm | 59 84 59 150 | C1, C3, Fz/mPFC, SMA, M1 | Increased gamma power at movement beginning |
| Ball et al. 2009 | ECoG | Center-out arm movements | 4 | Arm | 52 128 | M1 | Gamma power can be used for decoding movement direction |
| Bramson et al. 2018 | MEG | Approach-avoidance task | 45 | Hand | 60 90 | aPFC, M1 | Emotional control elicited increased PFC-M1 theta-gamma coupling |
| Bramson et al. 2020 | tACS | Approach-avoidance task | 44 | Hand | 75 | aPFC, M1 | Increased emotional control when gamma power is synchronized with peaks of aPFC theta activity |
| Brovelli et al. 2005 | sEEG | Motor preparation, working memory | 1 | Finger | 60 200 | PMC | Gamma power differentiates attention/memory and intentional tasks |
| Brown et al. 1998 | MEG | Sustained isometric contraction | 10 | Arm, foot | 30 60 | M1 | Increased CMC with muscular strength |
| Brown et al. 2001 | DBS | Tonic voluntary contractions | 4 | Arm | 70 | STN, GP | Increased gamma power and coherence by dopamine treatment in STN and GB in Parkinson patients |
| Brucke et al. 2008 | DBS | Go/No-Go task | 11 | Finger | 60 80 | GP | Increased contralateral gamma activity at the GP for motor responses |
| Brucke et al. 2012 | DBS | Choice reaction time | 22 | Arm | 35 105 | GP | Increased gamma power for larger and faster movements |
| Butorina et al. 2014 | MEG | Movement mirror illusion | 14 | --- | 55 85 | Left central | Increased gamma power in the mirror illusion |
| Cheyne et al. 2008 | MEG | Self-paced movements | 9 | Foot, finger, elbow | 60 90 | M1 | Increased gamma power activity for upper relative lower body parts |
| Cheyne and Ferrari 2013 | MEG | Self-paced movements | 9 | Foot, finger, elbow | 60 90 | M1 | Motor gamma is consistent within individuals and show differences between individuals |
| Cheyne et al. 2014 | MEG | Finger movements | 17 | Finger | 70 80 30 45 | M1 | Increased variability in the peak frequency of gamma in young children |
| Crone et al. 1998 | ECoG | Muscle contractions in response to body movement videos | 3,5 | Tongue, eye, hand, foot | 35 50 75 100 | M1 | Increased gamma power at movement beginning |
| Dalal et al. 2007 | MEG, ECoG | Self-paced movements | 12,2 | Finger | 65 90 | M1, cerebellum | Gamma sources can be reconstructed with MEG |
| Darvas et al. 2010 | EEG, ECoG | Finger movements (abduction/finger tap) | 10 | Finger | 83 101 | M1 | Focalized gamma power, a bi-phasic time course and bi-hemispheric phase-locking. PMC precede M1 activity |
| Darvas et al. 2013 | EEG | Passive observation | 10 | --- | 70 100 | M1 | Increased gamma power activity induced by movement observation |
| Djalovski et al. 2021 | EEG | Motor coordination, empathy task | 67 | Hand, whole body | 31 48 | Cz, C3, C4 | Distinct patters of neuro-behavioral coordination for two types of social interactions |
| Durschmid et al. 2014 | ECoG | Serial response, auditory motor coordination, Go/No-Go | 6 | Finger | 80 180 | M1, PMC, PFC | Increased performance is associated with an increment of theta-gamma coupling |
| Fang et al. 2009 | EEG | Shoulder flexion and elbow extensions | 12p,8h | Arm | 30 40 | Central, frontal, posterior | Reduced gamma CEC in stroke patients |
| Fischer et al. 2017 | DBS | Finger taping stoping task | 9 | Finger | 60 90 | STN | Increased gamma power for successful stopping |
| Fisher et al. 2020 | ECoG | Hand gripping | 12 | Hand | 60 80 | M1, STN | Increased spike-phase coupling for fast reaction times |
| Gaetz et al. 2010 | MEG | Brisk transient abductions | 10,20 | Finger | 60 90 | M1 | Increased gamma power in adolescence and decreased in adulthood |
| Gaetz et al. 2011 | MRS, MEG | Cued button presses | 9 | Finger | 60 90 | M1 | Increased beta power is associated with increased levels of GABA |
| Gaetz et al. 2013 | MEG | Multisource interference task | 24 | Finger | 60 90 | PMC, R-IFG | Increased gamma power for interference |
| Grent-t'-Jong et al. 2013 | MEG | Eriksen Flanker task | 15 | Finger | 60 90 | Central | Increased gamma power is evoked for interference |
| Gross et al. 2005 | MEG | Isometric contractions, wrist flexions and extensions | 10 | Arm | 26 40 | M1 | Increased CMC during isometric contractions |
| Gross et al. 2005 | MEG | Isometric contractions, wrist flexions and extensions | 10 | Arm | 26 40 | M1, SMA | Increased M1-M1 coupling for bimanual relative to unimanual movement and increased gamma CMC for hold movements |
| Grosse-Wentrup et al. 2011 | EEG | Motor imagery | 10 | --- | 55 85 | Central | Increased gamma power activity for motor imagery |
| Guo et al. 2012 | MEG | --- | 6p, 6h | Finger | 70 200 | S1, M1 | Increased ipsilateral gamma activity in children with cerebral palsy |
| Gwin et al. 2011 | EEG | Walking | 8 | Lower limbs | 50 150 | S1, M1, parietal and cingulate cortex | Increased gamma oscillations for walking |
| Gwin and Ferris 2012 | EEG | Isometric and isotonic knee and ankle movements | 8 | Lower limb | 31 45 | Left central | Increased gamma coherence for isotonic versus isometric movements |
| Hall et al. 2011 | MRS, MEG | Cued button presses | 8 | Finger | 60 90 | M1 | Gama power is not modulated by diazepam |
| Heinrichs-Graham. et al. 2018 | MEG | Eriksen Flanker task | 42 | Finger | 60 80 | M1 | Increased gamma frequency for interference |
| Hertz et al. 2012 | EEG | Extension-flexion, isometric contractions | 13 | Finger, foream | 31 48 | M1, PMC, SMA | Distinct patterns of M1-SMA coupling are evoked for distinct movements |
| Hoffman et al. 2019 | MEG | Eriksen Flanker task | 12p, 26h | Finger | 68 82 | M1 | Reduced gamma responses during motor execution in children with cerebral palsy |
| Huo et al. 2011 | MEG | Finger movements | 60 | Finger | 65 150 | M1 | Increased ipsilateral gamma power with age |
| Isabella et al. 2015 | MEG | Go/Switch/No-Go | 12 | Finger | 60 90 | M1 | Increased gamma power for switches |
| Joundi et al. 2012 | DBS | Arm reaching movements | 11 | Arm | 70 90 | STN | Increased gamma power is associated with faster movements |
| Kurz et al. 2014 | MEG | Knee movement task | 13p, 14h | Lower limb | 38 56 | S1, M1, parietal cortex | Reduced gamma activity in children with cerebral palsy |
| Leuthardt et al. 2004 | ECoG | Joystick control | 4 | Hand | 60 160 | S1, M1 | Gamma power can be used for decode movement direction |
| Li et al. 2020 | EEG | Unimanual and bimanual muscle contractions | 9 | Finger | 30 50 | Left central | Increased force lead to a shift beta-gamma in CMC |
| Lofredi et al. 2018 | DBS | Forearm pronation movements | 16 | Arm | 40 90 | STN | Increased gamma power for larger movements |
| Marsden et al. 2000 | ECoG | Phasic versus contractions | 6 | Arm | 61 100 | M1, S1 | CMC occurs for slow movements |
| Mehrkanoon et al. 2014 | EEG | Isometric finger movements | 12 | Finger | 30 80 | Left central | Increased gamma CMC during fast force transitions |
| Miller et al. 2007 | ECoG | Flexion and extension of all fingers or tongue | 22 | Finger, tongue | 76 100 | S1, M1 | Confirm somatotopic gamma activity for body parts |
| Miller et al. 2010 | ECoG | Motor imagery | 8 | --- | 76 100 | M1 | Increased gamma power induced by motor imagery |
| Mima et al. 1999 | EEG | Tonic contraction task | 8 | Fingers | 37 44 | Fronto-central | Increased CMC for the stronger isometric contraction |
| Mima et al. 2000 | EEG | Tonic contractions | 9 | Hand, arm, foot | 14 50 | Left central | Vibratory stimuli interference do not affect CMC |
| Muthukumaraswamy 2010 | MEG | Cued vs voluntary, isometric, active vs passive | 19 | Finger | 60 90 | M1 | Increased gamma power is associated with greater force |
| Muthukumaraswamy et al. 2011 | MEG | Ballistic, repetitive and near-isometric statics contractions | 6, 8, 10 | Finger | 60 90 | M1 | Increased gamma power without CMC increase for static force production |
| Muthukumaraswamy et al. 2013 | MEG, tiagabine | Cued finger movement task | 15 | Finger | 60 90 | M1 | Gamma power is not modulated by endogenous GABA |
| Nowak et al. 2017 | tACS, TMS | Go/No-Go task | 20 | Finger | 60 90 | M1 | Driving gamma oscillations in M1 produces a decrease of GABA inhibition |
| Oliveira et al. 2019 | EEG | Motor task | 11 | Arm | 31 90 | F3, C3 | Decreased gamma power for greater motor control |
| Omlor et al. 2007 | EEG | Static and dynamic contractions | 8 | Finger | 30 45 | Left central | Increased CMC for dynamics versus static movements |
| Pfurtscheller and Neuper 1992 | EEG | Self-paced movemens | 3 | Finger | 40 | Left central | Increased 40 Hz response for movements |
| Pfurtscheller et al. 1993 | EEG | Presses, flexion, touching | 1 | Finger, toe, tongue | 40 | Left central | Increased hand area, vertex and bi-lateral gamma activities are evoked for movements |
| Pfurtscheller et al. 2003 | ECoG | Hand movements (palmar pinch, tongue protrusions) | 4 | Hand, tongue | 65 95 | M1, parietal | Increased gamma power activity at the contralateral side |
| Rossiter et al. 2013 | MEG | Isometric hand grips | 25p, 23h | Arm | 30 80 | M1 | More scattered peaks of gamma CEC in stroke patients |
| Salenius et al. 1996 | MEG | Finger movements | 1 | Finger | 35 41 | M1 | Increased CMC for slow movements |
| Schoeffelen et al. 2005 | MEG | Wrist extensions | 6 | Hand | 40 47 | M1 | Increased CMC is related to readiness to respond |
| Schoeffelen et al. 2011 | MEG | Bimanual wrist extention task | 11 | Hand | 40 47 | M1 | Increased CMC is related to readiness to respond in both hands |
| Seebar et al. 2016 | MEG | Rhyhmic finger movements | 16 | Finger | 60 80 | M1, SMA | Increased and sustained gamma for repetitive movements |
| Short et al. 2020 | EEG | Walking | 9p, 12h | Lower limb | 25 50 | Frontal, central, posterior | Increased gamma responses during waking in children with cerebral palsy |
| Spooner et al. 2020 | MEG | Posner task | 63 | Finger | 60 80 | M1 | A motor gamma-theta network is involved in the reorientation of responses |
| Spooner et al. 2021 | MEG | Eriksen Flanker task | 69 | Finger | 72 84 | M1 | Gamma power predict RT increase for incongruent responses in young participants |
| Spooner et al. 2021 | MEG, CES | Unilateral flexion-extension to visual (clock-like) stimuli | 25 | Finger | 64 82 | M1 | Gamma-theta PAC in motor response is modulated by medium nerve stimulation |
| Swann et al. 2012 | ECoG | Maybe Stop/No Stop task | 1 | Finger | 70 250 | preSMA, IFG | Increased gamma power at the preSMA and then at the rIFG to prepare of a stop response |
| Szurhaj et al. 2005 | sEEG | Finger movements | 8 | Finger | 40 60 | M1, SMA, PMC | Increased gamma power at the beginning or the end of the movements |
| Tamas et al. 2018 | EEG | Contraction and brisk movements | 11 | Hand | 30 149 | S1, M1, SMA, dPFC, thalamus, cerebellum | Increased gamma coupling for finger contraction |
| Tan et al. 2013 | DBS | Manual grips | 9 | Hand | 55 90 101 375 | STN | Increased gamma power for greater effort |
| Tecchio et al. 2008 | MEG | Isometric contractions with fingers | 11 | Hand | 61 90 | M1, S1 | A combination of gamma activity at the M1 and S1 reflected performance |
| Trevanow et al. 2019 | MEG | Index presses to visual stimuli | 83 | Finger | 74 84 | M1 | In early adolescence gamma power becomes weaker with age |
| Wiesman et al. 2020 | MEG | Multisource interference task | 23 | Finger | 64 84 | M1, PMC | Increased gamma power is evoked for multisource interference |
| Wiesman et al. 2021 | MEG | Bimanual Go/No-Go task | 25 | Finger | 66 86 | M1 | Increased gamma power for uncertain versus certain contexts |
| Wilson et al. 2010 | MEG | Unilateral flexion-extension to visual (clock-like) stimuli | 10 | Finger | 74 86 | M1, SMA | Gamma power is more widespread in young humans. Gamma power decreases with age |
| Wilson et al. 2011 | MEG | Unilateral flexion-extension to visual (clock-like) stimuli | 12p, 10h | Finger | 74 86 | M1, SMA, cerebellum | Adolescents with early-onset psychosis shows decreased motor gamma activation |
| Wilson et al. 2011 | MEG | Unilateral flexion-extension to visual (clock-like) stimuli | 4 | Finger | 74 86 | M1 | Reduced gamma power after therapy with peripheral nerve stimulation in patients with stroke |
| Yanagisawa et al. 2012 | ECoG | Hand and elbow movements | 12 | Hand, arm | 80 150 | M1 | Gamma power contains information useful to decode movements |

For the N column. The number of participants is depicted. Two or more numbers indicate distinct set of participants. In clinical studies "p" stand for patient and "h" stand for healthy participant. For the Frequency column. The numbers indicate the frequency range. Two or more numbers indicate distinct frequency ranges reported.
